# Sexually Transmitted Infection Knowledge among Older Adults: Psychometrics and Test–Retest Reliability

**DOI:** 10.3390/ijerph17072462

**Published:** 2020-04-03

**Authors:** Matthew Lee Smith, Caroline D. Bergeron, Heather H. Goltz, Tammy Coffey, Ali Boolani

**Affiliations:** 1 Center for Population Health and Aging, Texas A&M University, College Station, TX 77843, USA; 2 School of Public Health, Texas A&M University, College Station, TX 77843, USA; 3 College of Public Health, The University of Georgia, Athens, GA 30602, USA; 4 Institut national de santé publique du Québec, Quebec, QC G1V 5B3, Canada; caroline.bergeron@inspq.qc.ca; 5 Baylor College of Medicine, University of Houston—Downtown, Houston, TX 77030, USA; goltzh@uhd.edu; 6 Alice Hyde Medical Center, Malone, NY 12953, USA; coffeytm@clarkson.edu; 7 Department of Physical Therapy and Department of Biology, Clarkson University, Potsdam, NY 13699, USA; aboolani@clarkson.edu

**Keywords:** aging, sexual health, sexual risk behavior, knowledge, measurement, scale validation

## Abstract

Sexually transmitted infections (STI) among older adults have dramatically increased in recent years, especially among those who are widowed and divorced. The purposes of this study were to: (1) identify STI-related knowledge among older adults; (2) report the psychometric properties of a tool commonly used to assess STI-related knowledge among younger populations using data from adults 65 years and older; and (3) determine test-retest reliability of the tool. Data were analyzed from 43 adults, aged 65–94 years, using the 27-item Sexually Transmitted Disease Knowledge Questionnaire (STD-KQ). Participants completed identical instruments on two separate days with approximately two weeks between. After responses were coded for correctness, composite scores were created. Cronbach’s reliability coefficients were calculated to determine response consistency, and Pearson’s r coefficients were used to assess test–retest reliability. Of 27 possible correct answers, participants reported an average of 11.47 (±6.88) correct responses on Day 1 and 11.67 (±7.33) correct responses on Day 2. Cronbach’s alpha coefficients for the 27-item composite scale were high for both days (0.905 and 0.917, respectively), which indicates strong response consistency. Pearson’s r coefficients were high between responses for the 27-item composite scale on Days 1 and 2 (*r* = 0.882, *p* < 0.01), which indicates strong test–retest reliability. Pearson’s r coefficients were high between responses for all but three of the 27 items when assessed separately. Findings suggest the utility of the STD-KQ to assess STI knowledge among older adults. However, the consistently low knowledge scores highlight the need for educational interventions among this population.

## 1. Introduction

Sexual activity is an important part of later life [1,2], frequently associated with better cardiovascular health [3,4], greater self-esteem [5], and higher life enjoyment levels [6]. While sexual activity levels typically decrease with age after 65 [7,8], large proportions of older adults desire sexual contact and engage in sexual activity [9,10,11]. Unfortunately, sexuality in later life is subject to stigma [12,13,14], misconceptions [15], and a reduced emphasis on safer sex behaviors [16,17], which all have led to unhealthy sexual practices increasing sexually transmitted infection (STI) rates among this age group.

According to the Centers for Disease Control and Prevention [18], STIs have more than doubled in the past ten years among U.S. adults age 65 years and older. The rates of primary and secondary syphilis have increased from 91 cases in 2007 to 349 cases in 2017, the rates for chlamydia have increased from 809 cases in 2007 to 2178 in 2017, and the rates for gonorrhea have increased from 707 cases in 2007 to 2063 cases in 2017 [18].

A lack of knowledge about STIs among older people may lead to existing misconceptions and inaccuracies [19,20,21,22,23]. Older adults have reported few opportunities to discuss their sexual health with their health care providers [24,25], which also limits their access to educational resources and interventions to reduce their STI risk [26].

Screening of STI knowledge may help foster sexual-health communication and interactions between older adults and their partners and healthcare providers, while also facilitating access to STI education for behavior change. There is no standardized or preferred tool to measure STI knowledge among adults over the age of 65. However, many validated instruments assess STI knowledge among younger adults, such as the Sexually Transmitted Disease Knowledge Questionnaire (STD-KQ) [27]. The purposes of this study were to: (1) report its psychometric properties for data collected among this older population; (2) determine the test–retest reliability of the tool; and (3) identify STI-related knowledge among older adults using the STD-KQ. After assessing available STI knowledge instruments, the STD-KQ was specifically selected because it was the most comprehensive in terms of STI types (bacterial and viral) and knowledge domains (symptomology, transmission, and treatment) [27]. We hypothesize that this tool will be just as effective in assessing STI knowledge among adults aged 65 years and older as it is for younger age groups. Additionally, we hypothesize that older adults will have low STI knowledge scores.

## 2. Materials and Methods

### 2.1. Population and Procedures

Older adults age 65 years and older were recruited for study participation from nursing homes, adult residential facilities, and elderly care centers located within St. Lawrence County, New York. Prior to recruitment, the research team contacted facility management to obtain permission and ensure compliance with residential standards and community culture. A total of 14 sites were approached, with four sites agreeing to participate. Administration promoted the data collection activity to their residents (i.e., sharing the date, time, and on-site location at their discretion) and encouraged their voluntary participation. Once on-site, the research team described the study in small group sessions. During the sessions, investigators explained the purpose of the study, what participants would be asked to do, and the questionnaire content and format. Investigators also answered any study-related questions from the older adults. To complement these recruitment efforts in residential facilities, a broader effort was undertaken to recruit community-dwelling older adults. Flyers were posted in the community, and the study was promoted by a local aging coalition. Potential participants were provided the contact information for study research assistants so they could schedule a time for data collection. Given the passive recruitment methods used to recruit residential facility residents and community-dwelling older adults, participation rates could not be calculated.

Older adults who expressed interest in participating in the study then completed individual Mini-Mental State Examinations (MMSE) with a research team member in a private, distraction-reduced environment. Those who scored between 30 and 24 on the MMSE were considered to have a score within normal range, indicating no cognitive impairment [28]. Individuals scoring lower than 24 were ethically unable to consent to the research, and therefore informed of their ineligibility. Following the MMSE, a written informed consent was obtained from each participant for the two-day study. All study procedures were approved by the Clarkson University Institutional Review Board (protocol # 19–16).

On Study Day One, qualified participants were assigned a randomized five-digit identification number. This five-digit number was used by participants to enter the online questionnaire using a survey delivery platform (surveymonkey.com) on laptops provided by the research team. Participants then completed the 27-item STD-KQ and a brief sociodemographic questionnaire. Upon completion of the online questionnaire, participants were asked to provide their personal telephone number so they could be contacted for Study Day Two. Participant telephone numbers were recorded and stored on a private password-protected computer. At the conclusion of Study Day One, participants were instructed not to discuss the study or instrument with others. They were also instructed not to attempt to locate correct answers independently. Rather, participants were told they would receive the correct responses to any of their questions after data collection on Study Day Two.

Study Day Two occurred approximately two weeks after Study Day One. Participants were asked to complete the STD-KQ using an online survey delivery platform in their respective nursing home, adult residential facility, or elderly care center. Participants were instructed to input the five-digit identification number they received on Study Day One and complete the questionnaire. At the conclusion of Study Day Two, participants were able to ask physician assistant trainees content-related questions regarding the survey. Participants were also given a pamphlet about safer sex practices, created by the New York State Department of Health.

A total of 54 participants were recruited into the study, of which one was omitted based on their MMSE score. Of the 53 participants who completed Study Day One, 10 were lost to follow-up. A total of 43 participants completed Study Day Two. Thus, 81.13% of recruited study participants completed both Study Days One and Two.

### 2.2. Measures

Knowledge. Participants completed the 27-item Sexually Transmitted Disease Knowledge Questionnaire (STD-KQ) [27]. Each item provided a statement about a sexually transmitted disease, and participants were instructed to indicate if the statement was true or false. Participants were also instructed not to guess about an answer; rather, indicate they don’t know. Items were scored as correct (score = 1) and incorrect (score = 0). Responses of don’t know were scored as incorrect. Composite scores of the total number of items correct were also calculated (scores ranging from 0 to 27 correct items).

Sociodemographics. Participants were asked to report their age, sex, race, education level, relationship status, and sexual identity on a brief sociodemographic questionnaire.

### 2.3. Statistical Analyses

All statistical analyses were performed in IBM SPSS Statistics, version 25 (Armonk, United States). Frequencies were computed for all sociodemographics and SDT-KQ items for both study days. The frequency of don’t know responses were reported for all 27 items for both study days. Furthermore, the frequency of correct responses (don’t know scored as incorrect) were reported for all 27 items for both study days. Cronbach’s internal reliability coefficients were calculated to determine response consistency across the 27 items separately for each study day. Pearson’s r coefficients were used to assess test–retest reliability across study days for each item and composite score. Similarly, Kappa statistics were used to examine the strength of concordance across study days for each item and composite score.

## 3. Results

The average age of the 43 participants was 76.48 (±9.01) years, with ages ranging from age 65 to 94 years. Most participants were female (74.4%) and Caucasian (97.7%). About 42% of participants reported having a high school diploma or less, and 16.3% reported having a 4-year college degree or post-graduate education. Approximately 28% of participants were married, 27.9% were widowed, and 27.9% were divorced. Approximately 70% of participants identified as heterosexual and 25.6% reported being celibate.

As presented in Table 1, of 27 possible correct answers, participants reported an average of 11.47 (±6.88) correct responses on Study Day One and 11.67 (±7.33) correct responses on Study Day Two. Cronbach’s alpha coefficients for the 27-item composite scale were high for both days (0.905 and 0.917, respectively), which indicates strong internal consistency. Pearson’s r coefficients were high between responses for the 27-item composite scale on Study Days One and Two (*r* = 0.882, *p* < 0.01), which indicates strong test–retest reliability. Pearson’s r coefficients were statistically significant for 26 of the 27 items (range = 0.306–0.701), when assessed separately. Similarly, Kappa statistics indicating agreement between responses from Study Days One and Two were statistically significant for the 27-item composite scale (Kappa = 0.102, *p* < 0.01) and 26 of the 27 items (range = 0.302–0.685), when assessed separately.

When examining the raw responses to the 27 STD-KQ items, the proportion of participants reporting they don’t know the correct answer ranged from 23.3% to 62.8% on Study Day One. For 12 of the 27 items, more than 40% of participants reported they don’t know the correct answer on Study Day One (44.4% of items). The proportion of participants reporting they don’t know the correct answer ranged from 25.6% to 53.5% on Study Day Two. For 8 of the 27 items, more than 40% of participants reported they don’t know the correct answer on Study Day Two (29.6% of items). On both days, the items receiving the most don’t know responses were related to viral infections including human papillomavirus (HPV) and human immunodeficiency virus (HIV).

After dichotomizing the 27 STD-KQ items into correct and incorrect responses, the proportion of participants who correctly answered items ranged from 23.3% to 76.7% on Study Day One. For 13 of the 27 items (48.2% of items), less than 40% of participants correctly answered these items on Study Day One. The proportion of participants who correctly answered these items on Study Day Two ranged from 18.6% to 67.4%. For 15 of the 27 items (55.6% of items), less than 40% of participants correctly answered these items on Study Day Two, Their barriers to condom use and reported fewer occasions of unprotected sex compared to participants in the control group. (Table 1).

## 4. Discussion

Given the increasing incidence and prevalence of STIs among older adults [18], it is important to assess their knowledge of STIs to promote sexual health and reduce sexual risk. Specific groups of sexually active older adults may be at increased risk of STIs. Those who are divorced or widowed [29,30,31,32] or those living in congregate settings [33] report increased sexual activity, often accompanied with feelings of freedom and openness to sexual experimentation [34,35,36], which may increase their risks of contracting STIs.

A study by Schick and colleagues [37] reported that substantial numbers of men (23.5%) and women (13.6%), aged 50 years and older, drank alcohol or had partners who drank prior to their most recent penile–vaginal intercourse. In that same study, almost 15% of men and 9% of women reported having sex with transactional partners or new acquaintances. Less than one quarter of all participants reported using condoms, even though more than 5% revealed their partners had an STI at their most recent sexual event. Given this population is relatively safe from unintended pregnancies, inaccurate appraisals of STI risk may stem from lack of sexual knowledge and underestimates of sexual risk for self and partners [38]. Screening for STI knowledge is the first step to providing comprehensive education on safer sex practices.

However, in their systematic review, Levy and collaborators [17] reported that the majority (89%) of STI risk-reduction clinical trials excluded people over the age of 65. Since then, MacDonald and colleagues [39] conducted a systematic review specifically on condom use interventions for middle-aged and older adults and identified only one effective randomized controlled trial for increasing condom use behavior as a prevention strategy for STI transmission. In Lovejoy and colleagues’ trial [40], through four one-on-one telephone sessions of about 40–50 min each with a Master’s degree level clinical psychology trainee, older participants in the intervention group were able to address Age-appropriate, evidence-based prevention education programs and interventions should be developed to support greater STI knowledge among older adults and improved sexual-health behaviors. Such programs will need rigorously validated and reliable tools to aid in identifying potential at-risk older adults. To achieve this aim, the current study used the STD-KQ, which is typically administered to younger adults, to assess its reliability for screening STI knowledge among older adults. Current findings suggest that the STD-KQ is a reliable and useful tool to assess STI knowledge of the older adult population. In fact, the tool revealed that a large proportion of the study sample of older adults lacked knowledge of STI risks, presentation, symptomology, transmission, or treatment. Participants had the lowest scores on items related to HPV, a STI that was not well known until the mid-1980s [41], but infects large swaths of the population who are sexually active at some point in their lives [42]. Low STI knowledge among older adults highlights the need for comprehensive system-level solutions.

In her integrative review of HIV-related risks in older adulthood, Savasta [43] found that 88% of the articles reported that “system failures,” such as lack of patient-provider communication and insufficient education programs, were associated with increased prevalence of HIV among older adults. As first-line responders in STI and HIV/AIDS prevention, physicians can be most effective by proactively assessing older patients’ STI knowledge during periodic health exams and initiating routine discussions regarding sexual-health matters [44,45,46,47,48]. Yet, there are several known barriers to physicians engaging in these patient-provider discussions, including not understanding the importance of sexuality in later life [46]; having limited communication training skills, educational materials, and self-efficacy to talk about sexual health with older patients [24,45,48]; and lacking time to initiate these discussions [49].

To prevent potential system failures, physicians could refer their older patients to sexual medicine professionals, including clinical social workers, sex educators, and sex therapists, who have more knowledge, educational and psychosocial resources, and who feel more comfortable engaging in conversations about STI prevention [24]. These clinical providers can use available evidence-based resources to educate their older patients, including the CDC’s ready-to-use STI curriculum (http://www2a.cdc.gov/stdtraining/ready-to-use), U.S. Department of Health & Human Services’ Office on Women’s Health (www.womenshealth.gov), or the University of Washington Center for AIDS and STD training resources (http://depts.washington.edu/cfas/training) [26]. Health care providers could also refer their older patients to educational opportunities and interventions to enhance knowledge and promote sexual health such those available through the Sexual Medicine Society of North America’s Sex Health Matters website (www.sexhealthmatters.org), The Fenway Institute/The National LGBT Health Education Center (https://www.lgbthealtheducation.org), and the American Geriatrics Society’s Health in Aging Foundation (https://www.healthinaging.org/a-z-topic/sexual-health).

### Limitations

The current study had a few limitations. Reliability of the STD-KQ was assessed using a small sample of older adults who were mostly white, female, heterosexual, from a congregate setting, and from one rural area in a single state. Considering that sexual health and behavior during older adulthood can be sensitive topics [12,13,14], it is possible that self-selection bias was introduced in our study. It may be that participants who enrolled were those who were particularly interested in the topic, sexually active, or only those who felt comfortable talking about sex. Furthermore, given 10 of the residential facilities approached refused to participate in the study, the underlying stigma and implicit biases related to these topics at the administration level may hinder sexuality research among older adults in residential facilities. Additional details about study promotion, interest, and refusal among facility residents and community-dwelling older adults were limited and hindered our ability to assess participation rates and the possible self-selection bias associated with this study. In residential facilities, non-participation and attrition rates from Study Day One to Study Day Two may reflect the administration’s diligence in promoting the study or the residents having schedule conflicts at the designated data collection times. Although participants were asked not to discuss the study topics and content or seek out correct answers between Study Days One and Two, it is possible that some form of contamination occurred.

Given the limited sample size, we were unable to make meaningful comparisons of STI knowledge across subgroups. Recent studies have demonstrated age- and health-related differences between community-dwelling older adults and those living in residential facilities [50,51]. When considered with recent attention to STI outbreaks in congregate living settings for older adults [52], a compelling argument for study replication emerges. Replicating this study with a larger and more robust sample from other settings and locations would allow for meaningful statistical comparisons of older adults with inadequate STI knowledge based on sociodemographic characteristics with known associations to both low sexual-health knowledge and high-risk sexual behaviors (e.g., relationship status, education level, or race/ethnicity).

Additionally, while the older adults in this sample exhibited low levels of STI knowledge (i.e., an average of 40% and 55% correct responses on Study Days One and Two, respectively), there is no recommended STD-KQ cut-point or threshold for insufficient knowledge among older adults. While such a score would be essential for informing healthcare professionals and providers concerning potential knowledge deficits, we did not make such a recommendation in the current study because of our sample size and homogeneous sample. Additionally, the STD-KQ was developed for evaluating knowledge of STIs in younger populations. Therefore, it may not include topics that are important for sexuality and have implications for STI risk in later life, such as intimacy, communication, companionship, changes in sexual functioning, and sexual consent in the absence or presence of cognitive decline [36,53,54,55,56]. The instrument also included some topics that may not be as relevant to an older demographic, including childbirth-related HIV transmission and HPV [based on Pap test and vaccination standards]). Adaptions to the STD-KQ may be warranted to take into account contextual factors influencing sexuality among older adults [36,53,54,55,56]. Lastly, while the instrument included a sexual identity item developed by the research team, this item may not have fully captured the possible range of responses. Future studies should use validated and more commonly used items for sexual identity and sexual orientation.

## 5. Conclusions

Screening for STI knowledge is an important first step to physicians providing or initiating referrals for comprehensive education on safer sex practices. The STD-KQ scale can be a useful tool for diverse types of medical and healthcare professionals to screen their older patients and subsequently initiate discussions on STI and HIV/AIDS prevention and sexual health.

## Figures and Tables

**Table 1:** Correct responses by testing day, test-retest reliability, and kappa statistics (n = 43).

		*Don’t Know Response n (%)*		*Correct Response n (%)*		*Test-Retest*	*Agreement*
Item #	Item Text	Day 1	Day 2	Day 1	Day 2	Pearson r	Kappa
1	Genital Herpes is caused by the same virus as HIV	19 (44.2%)	17 (39.5%)	12 (27.9%)	16 (37.2%)	0.701 **	0.685 **
2	Frequent urinary infections can cause Chlamydia	22 (51.2%)	19 (44.2%)	12 (27.9%)	15 (34.9%)	0.306 *	0.302 *
3	There is a cure for Gonorrhea	12 (27.9%)	13 (30.2%)	26 (60.5%)	28 (65.1%)	0.606 **	0.603 **
4	It is easier to get HIV if a person has another Sexually Transmitted Disease	12 (27.9%)	13 (30.2%)	20 (46.5%)	17 (39.5%)	0.581 **	0.575 **
5	Human Papillomavirus (HPV) is caused by the same virus that causes HIV	27 (62.8%)	22 (51.2%)	11 (25.6%)	15 (34.9%)	0.466 **	0.454 **
6	Having anal sex increases a person’s risk of getting Hepatitis B	25 (58.1%)	14 (32.6%)	11 (25.6%)	20 (46.5%)	0.415 **	0.374 **
7	Soon after infection with HIV a person develops open sores on his or her genitals (penis or vagina)	18 (41.9%)	13 (30.2%)	10 (23.3%)	8 (18.6%)	0.161	0.160
8	There is a cure for Chlamydia	21 (48.8%)	18 (41.9%)	19 (44.2%)	24 (55.8%)	0.509 **	0.495 **
9	A woman who has Genital Herpes can pass the infection to her baby during childbirth	10 (23.3%)	11 (25.6%)	33 (76.7%)	29 (67.4%)	0.440 **	0.428 **
10	A woman can look at her body and tell if she has Gonorrhea	19 (44.2%)	18 (41.9%)	17 (39.5%)	18 (41.9%)	0.374 *	0.374 *
11	The same virus causes all of the Sexually Transmitted Diseases	13 (30.2%)	11 (25.6%)	27 (62.8%)	27 (62.8%)	0.403 **	0.403 **
12	Human Papillomavirus (HPV) can cause Genital Warts	24 (55.8%)	23 (53.5%)	13 (30.2%)	16 (37.2%)	0.436 **	0.413 **
13	Using a natural skin (lambskin) condom can protect a person from getting HIV	14 (32.6%)	13 (30.2%)	12 (27.9%)	9 (20.9%)	0.572 **	0.562 **
14	Human Papillomavirus (HPV) can lead to cancer in women	19 (44.2%)	16 (37.2%)	21 (48.8%)	24 (55.8%)	0.495 **	0.490 **
15	A man must have vaginal sex to get Genital Warts	14 (32.6%)	13 (30.2%)	19 (44.2%)	14 (32.6%)	0.581 **	0.564 **
16	Sexually Transmitted Diseases can lead to health problems that are usually more serious for men than women	14 (32.6%)	14 (32.6%)	18 (41.9%)	21 (48.8%)	0.397 **	0.393 **
17	A woman can tell that she has Chlamydia if she has a bad smelling odor from her vagina	17 (39.5%)	20 (46.5%)	10 (23.3%)	13 (30.2%)	0.597 **	0.587 **
18	If a person tests positive for HIV the test can tell how sick the person will become	12 (27.9%)	11 (25.6%)	25 (58.1%)	25 (58.1%)	0.522 **	0.522 **
19	There is a vaccine available to prevent a person from getting Gonorrhea	21 (48.8%)	17 (39.5%)	16 (37.2%)	17 (39.5%)	0.657 **	0.656 **
20	A woman can tell by the way her body feels if she has a Sexually Transmitted Disease	16 (37.2%)	14 (32.6%)	18 (41.9%)	14 (32.6%)	0.618 **	0.606 **
21	A person who has Genital Herpes must have open sores to give the infection to his or her sexual partner	10 (23.3%)	13 (30.2%)	16 (37.2%)	12 (27.9%)	0.486 **	0.476 **
22	There is a vaccine that prevents a person from getting Chlamydia	22 (51.2%)	21 (48.8%)	16 (37.2%)	14 (32.6%)	0.595 **	0.591 **
23	A man can tell by the way his body feels if he has Hepatitis B	17 (39.5%)	17 (39.5%)	20 (46.5%)	17 (39.5%)	0.486 **	0.481 **
24	If a person had Gonorrhea in the past he or she is immune (protected) from getting it again	10 (23.3%)	13 (30.2%)	31 (72.1%)	28 (65.1%)	0.415 **	0.409 **
25	Human Papillomavirus (HPV) can cause HIV	27 (62.8%)	20 (46.5%)	11 (25.6%)	15 (34.9%)	0.689 **	0.673 **
26	A man can protect himself from getting Genital Warts by washing his genitals after sex	15 (32.9%)	12 (27.9%)	24 (55.8%)	24 (55.8%)	0.529 **	0.529 **
27	There is a vaccine that can protect a person from getting Hepatitis B	13 (30.2%)	12 (27.9%)	25 (58.1%)	22 (51.2%)	0.402 **	0.393 **
	Composite Score of Total Correct: Average (range from 0 to 27)			11.47 (±6.88)	11.67 (±7.33)	0.882 **	0.102 **
	Composite Score of Total Correct: Cronbach’s Alpha (based on standardized items)			0.905	0.917		

* *p* < 0.05; ** *p* < 0.01
